# Identification of subgroup effect with an individual participant data meta-analysis of randomised controlled trials of three different types of therapist-delivered care in low back pain

**DOI:** 10.1186/s12891-021-04028-8

**Published:** 2021-02-16

**Authors:** Siew Wan Hee, Dipesh Mistry, Tim Friede, Sarah E. Lamb, Nigel Stallard, Martin Underwood, Shilpa Patel, On behalf of the Repository Group, Christer Carlsson, Francesca Cecchi, Ninna Dufour, Heinz Endres, Mark Hancock, Elaine Hay, Von Korff, Sarah Lamb, Luciana Macedo, Hugh MacPherson, Chris Maher, Suzanne McDonough, Rob Smeets, David Torgerson, Claudia Witt

**Affiliations:** 1 Statistics and Epidemiology Unit, Warwick Medical School, University of Warwick, Coventry, CV4 7AL UK; 2 Warwick Clinical Trials Unit, Warwick Medical School, University of Warwick, Coventry, CV4 7AL UK; 3 Department of Medical Statistics, University Medical Center Göttingen, 37073 Göttingen, Germany; 4 University of Exeter Medical School, St Lukes Campus, Heavitree Road, Exeter, EX1 2LU UK; 5 University Hospitals of Coventry and Warwickshire, Coventry, UK

**Keywords:** Low back pain, Stratification, Subgroups, IPD, Therapist delivered interventions, Physical interventions, Psychological interventions

## Abstract

**Background:**

Proven treatments for low back pain, at best, only provide modest overall benefits. Matching people to treatments that are likely to be most effective for them may improve clinical outcomes and makes better use of health care resources.

**Methods:**

We conducted an individual participant data meta-analysis of randomised controlled trials of three types of therapist delivered interventions for low back pain (active physical, passive physical and psychological treatments). We applied two statistical methods (recursive partitioning and adaptive risk group refinement) to identify potential subgroups who might gain greater benefits from different treatments from our individual participant data meta-analysis.

**Results:**

We pooled data from 19 randomised controlled trials, totalling 9328 participants. There were 5349 (57%) females with similar ratios of females in control and intervention arms. The average age was 49 years (standard deviation, SD, 14).

Participants with greater psychological distress and physical disability gained most benefit in improving on the mental component scale (MCS) of SF-12/36 from passive physical treatment than non-active usual care (treatment effects, 4.3; 95% confidence interval, CI, 3.39 to 5.15). Recursive partitioning method found that participants with worse disability at baseline gained most benefit in improving the disability (Roland Morris Disability Questionnaire) outcome from psychological treatment than non-active usual care (treatment effects, 1.7; 95% CI, 1.1 to 2.31). Adaptive risk group refinement did not find any subgroup that would gain much treatment effect between psychological and non-active usual care. Neither statistical method identified any subgroups who would gain an additional benefit from active physical treatment compared to non-active usual care.

**Conclusions:**

Our methodological approaches worked well and may have applicability in other clinical areas. Passive physical treatments were most likely to help people who were younger with higher levels of disability and low levels of psychological distress. Psychological treatments were more likely to help those with severe disability. Despite this, the clinical importance of identifying these subgroups is limited. The sizes of sub-groups more likely to benefit and the additional effect sizes observed are small. Our analyses provide no evidence to support the use of sub-grouping for people with low back pain.

**Supplementary Information:**

The online version contains supplementary material available at 10.1186/s12891-021-04028-8.

## Background

Low back pain (LBP) is the leading cause of disability globally, with an increasing burden [1]. Stratified care, delivering the right treatment to the right person at the right time, could potentially reduce this burden [2, 3]. Conducting randomised controlled trials (RCTs) to identify subgroups who benefit from particular treatments to inform stratification is challenging. Typically, in the UK, a good quality RCT costs £1-2 m and takes up to 5 years.

The standard approach to subgroup identification is to measure effect moderation of baseline variables in an interaction analysis [4]. The interaction analysis estimates the response effect where the baseline characteristic of interest moderates the treatments. Substantially larger numbers are needed to show these moderation effects than are needed to show main treatment effects of the same magnitude [5]. A systematic review of subgroup analyses in LBP trials found the overall quality to be poor [6] with few studies having statistical power to detect realistic moderation effects. Furthermore, standard approaches consider one factor at a time. Combinations of factors might identify clinically recognisable subgroups with larger moderation effects. The use of individual participant data (IPD) meta-analysis of RCTs may provide power to identify subgroups defined by multiple factors benefiting most from particular treatments.

A 2019 IPD meta-analysis (*m* = 27 trials, *n* = 3514 participants) of exercise therapy for low back pain (LBP) found a small number of statistically significant characteristics that moderated treatment outcomes [7].

As part of a National Institute for Health Research programme grant we developed a repository of data from RCTs of therapist delivered active physical, passive physical and psychological interventions for LBP published between 1999 and 2012 [8, 9]. For brevity, the term therapist-delivered interventions include non-pharmacological interventions delivered by therapists including physiotherapists, occupational therapists, chiropractors, osteopaths and psychologists. Our aim was to understand which participants are most likely to benefit from which treatment approaches to help improve the clinical and cost effectiveness of future LBP treatments. In this programme of work, we developed two different approaches to subgroup identification and used these approaches to estimate the magnitude of the identifiable subgroup effects. This paper presents the results of applying these two statistical approaches.

## Methods

Full details of the programme are published [9] elsewhere. Here we summarise part of the programme of work. Ethical approval was granted by Oxford Central REC (11/SC/0232).

### Identifying the data and developing a pooled repository

We did a systematic review to identify potential moderators to apply to our dataset. The studies identified in this review formed the basis of the trials we sought to include in this study [10]. In this review MEDLINE, EMBASE, Web of Science and Citation Index and Cochrane Controlled Trials Register (CENTRAL) databases were searched using the terms ‘low back pain’ combined with ‘trial’, ‘observational’, ‘cohort’ and ‘prospective studies’. Two independent reviewers assessed risk of bias based on these criteria: method of randomisation, allocation concealment, incomplete outcome data, selective outcome reporting, and other sources of bias. We searched the original search output for randomised control trials that had interventions being delivered by a therapist and had a sample size > 179. We invited investigators of the trials identified to share trial data with us. We focussed on recently published larger trials to ensure we included higher quality studies, where data would be more likely to be available. Including large numbers of small studies would have substantially increased work needed to prepare data for inclusion in our database. Having said this we were offered data from a few smaller studies which we decided to include to improve the statistical power of our analysis. Full details of our approach to obtaining data and developing and managing the repository are published elsewhere [8].

As trials had a range of therapist-delivered and control interventions we grouped this to allow meaningful analysis. Using a similar approach to the American Pain Society/American College of Physicians guidelines of grouping non-pharmacological interventions [11], groups were: control (non-active usual care), sham control (sham acupuncture, electrotherapy, advice/education, mock transcutaneous electrical nerve stimulation), active physical (exercise and graded activity), passive physical (individual physiotherapy, manual therapy, acupuncture) and psychological (advice/education, psychological therapy) [8, 9].

As trials had different follow-up times, we classified follow-up into short- (2 and 3 months), mid- (6 months) and long-term (12 months post randomisation). We classified the 32 patient reported outcome measures (PROMs) used into physical disability, pain, psychological distress and non-utility quality of life. As has previously been shown, LBP disability measures cannot be mapped into a single outcome [12], analyses were therefore only performed on measures common to more than one trial. We have presented the response for each clinical outcome measurement as the change from baseline to the follow-up time point with a positive score representing an improvement. Individual items (if available) were used to obtain the composite score otherwise, the original individual composite scores were used.

### Descriptive analysis

We summarised categorical data as frequency and percentage, and continuous data as mean and standard deviation (SD), by treatment arm; control (non-active usual care and sham) and intervention (active physical, passive physical, psychological, and combination). Our main analyses were based on complete case analysis with missing data due to non-responders or withdrawals not imputed. Analyses were performed on IPD from at least two trials so as not to replicate original analyses.

### Identification of moderators

We identified potential moderators in two ways. Firstly, from our systematic review identifying potential treatment moderators (factors measured pre-randomisation indicating who benefits most and least from a treatment) [10]. Secondly, including IPD from all RCTs in a single mixed-effects meta-analysis model for each follow-up time with moderators declared statistically significant at the two-sided 5% or weakly significant at the two-sided 20% level [13].

### Approaches to subgroup identification

We applied two approaches to identification of subgroups: Recursive Partitioning (RP) [14] and Adaptive Refinement by Directed Peeling (ARDP) [15]. Both aim to identify subgroups of participants with treatment effect larger than for other participants, by considering subgroups defined by ranges of values for sets of moderators. The RP method creates subgroups by successively splitting the population to build up a subgroup. It utilises a splitting criterion to create binary splits of the covariate space thus forming a tree-like structure. This splitting criterion isthe *p*-value of the subgroup effect (treatment by covariate interaction) which is estimated using a mixed-effects model to account for the between trial heterogeneity.

The ARDP method starts with the whole population then removes parts of it, thereby increasing the observed treatment effect in the remaining subgroups. The criterion for optimisation is based on the interaction between treatment and subgroup which allows for between-trial heterogeneity. This method splits categorical covariates using each of its categories, for example, sex would be split into male and female. Therefore, categorical covariates with three or fewer categories would cause the method to remove a large proportion of participants at each stage, an unappealing feature. Covariates with three or fewer categories were not included in this analysis.

To establish proof of principle for our novel methods we first ran our analyses on the overall dataset before running our main analyses for the pairwise comparisons of active physical, passive physical and psychological treatments against control. It is these three distinct comparisons that are the clinically outputs from this study. We present our methodological steps in some detail to introduce the reader to our methodological approach.

## Results

### Descriptive and one-step meta-analysis

We collected data from 9328 participants from 19 trials (Tables 1 and 2). We identified three broad treatment types within the data repository for which we wish to explore potential moderators; (i) active physical, ii) passive physical, iii) psychological treatments. Control arms included non-active usual care and sham intervention.

**Table 1:** Included trials

Given name of trial	Country	Number of participants	Grouping of treatments	Interventions tested
Witt [16]	Germany	3093	Passive physical	Acupuncture
			Control	Usual/GP care control
UK BEAM [17]	United Kingdom	1334	Active physical	Exercise
			Passive physical	Manipulation
			Combination	Combination (exercise + manipulation)
			Control	Usual/GP care control
Haake [18]	Germany	1163	Passive physical	Acupuncture
			Control	Usual/GP care control
			Sham control	Sham acupuncture
BeST [19]	United Kingdom	701	Psychological	Psychological plus exercise/activities
			Control	Usual/GP care control
Keele [20]	United Kingdom	402	Passive physical	Manipulation
			Psychological	Psychological
Brinkhaus [21]	Germany	298	Passive physical	Acupuncture
			Control	Usual/GP care control
			Sham control	Sham acupuncture
Dufour [22]	Denmark	286	Passive physical	Individual physiotherapy
			Active physical	Exercise
Pengel [23]	Australia	260	Active physical	Exercise
			Psychological	Advice/education
			Combination	Combination (exercise + education)
			Sham control	Sham electrotherapy + education
YACBAC [24]	United Kingdom	241	Passive physical	Acupuncture
			Control	Usual/GP care control
Hancock [25]	Australia	240	Passive physical	Manipulation
			Sham control	Sham electrotherapy
Von Korff BIA [26]	United States of America	240	Psychological	Psychological
			Control	Usual/GP care control
HullExPro [27]	United Kingdom	237	Passive physical	Individual physiotherapy
			Active physical	Exercise
Von Korff SC2 [28]	United States of America	226	Psychological	Psychological
			Control	Usual/GP care control
Smeets [29]	The Netherlands	223	Active physical	Exercise
			Active physical	Graded activity
			Combination	Combination (education + graded activity)
			Control	Usual/GP care control
Cecchi [30]	Italy	210	Passive physical	Individual physiotherapy
			Passive physical	Manipulation
			Psychological	Advice/education
York BP [31]	United Kingdom	187	Active physical	Exercise
			Control	Usual/GP care control
Macedo [32]	Australia	172	Psychological	Advice/education
			Active physical	Graded activity
Carlsson [33]	Sweden	50	Passive physical	Acupuncture
			Sham control	Sham TENS
Kennedy [34]	United Kingdom	48	Passive physical	Acupuncture
			Sham control	Sham TENS

**Table 2:** Demographic details

Characteristics	Control^**c**^ (No. of trials ***m*** = 14; No. of participants ***n*** = 3573)		Intervention^**d**^ (No. of trials ***m*** = 19; No. of participants ***n*** = 5755)		All (No. of trials ***m*** = 19; No. of participants ***n*** = 9328)	
***Demographics***						
*Age, years*						
No. of trials, *m*: no. of participants, *n*	14	3573	19	5753	19	9326
Mean (SD)	50.13	(13.77)	48.18	(13.9)	48.92	(13.88)
*Sex*						
No. of trials, *m*	14		19		19	
Female (%)	2053	(57.47)	3296	(57.28)	5349	(57.36)
Male (%)	1519	(42.53)	2458	(42.72)	3977	(42.64)
*Ethnicity*						
No. of trials, *m*	4		5		5	
White (%)	478	(89.35)	891	(88.66)	1369	(88.9)
Mixed (%)	3	(0.56)	4	(0.40)	7	(0.45)
Black (%)	21	(3.93)	26	(2.59)	47	(3.05)
Asian (Indian, Pakistani, Bangladeshi, others) (%)	17	(3.18)	44	(4.38)	61	(3.96)
Chinese (%)	1	(0.19)	2	(0.20)	3	(0.19)
Others (%)	15	(2.80)	38	(3.78)	53	(3.44)
*Smoking status*						
No. of trials, *m*	2		6		6	
No (%)	123	(74.10)	763	(64.39)	886	(65.58)
Yes (%)	43	(25.90)	422	(35.61)	465	(34.42)
*Employment status*						
No. of trials, *m*	7		11		11	
Full time employment (%)	489	(53.80)	1256	(49.68)	1745	(50.77)
Part time employment (%)	190	(20.90)	442	(17.48)	632	(18.39)
No employment (%)	230	(25.30)	830	(32.83)	1060	(30.84)
*BMI*						
No. of trials, *m*: no. of participants, *n*	2	915	5	1189	5	2104
Mean (SD)	26.44	(4.60)	1189	(26.67)	26.57	(4.73)

^a^m, number of trials

^b^n, number of participants

^c^Control arm includes best usual care and sham treatments. Four trials did not have control arm

^d^Intervention arm includes active physical, passive physical, psychological and combination

There were 5349 (57%) females with similar ratios of females in control and intervention arms. The average age was 49 years (standard deviation, SD, 14). The age range is slightly different across treatment arms due to different inclusion criteria of the trials [9].

The most frequently used PROM for physical disability was the Roland Morris Disability Questionnaire (RMDQ), (*m* = 14 trials, *n* = 4710 participants). This was followed by the disability score domain in Chronic Pain Grade (CPG-DS) (*m* = 4, *n* = 3328), the Hannover functional ability questionnaire for measuring back-pain related functional limitations (FFbHR) (*m* = 3, *n* = 4176) and the patient specific functional scale (PSFS) (*m* = 3*, n* = 667) (Additional file 1: Appendix 1). The physical disability, functional limitation and pain mean scores between control and intervention arms at baseline were very similar. The mean RMDQ score was 9.9 (SD, 5.1; where a maximum score of 24 was worst), CPG-DS was 50.2 (SD, 22; where a maximum score of 100 was worst), and FFbHR was 57.6 (SD, 20.5; where a maximum score of 100 was best). Most trials measured psychological distress but the wide variety patient reported outcome measures (PROMs) made direct comparisons impossible.

**Fig. 1:**
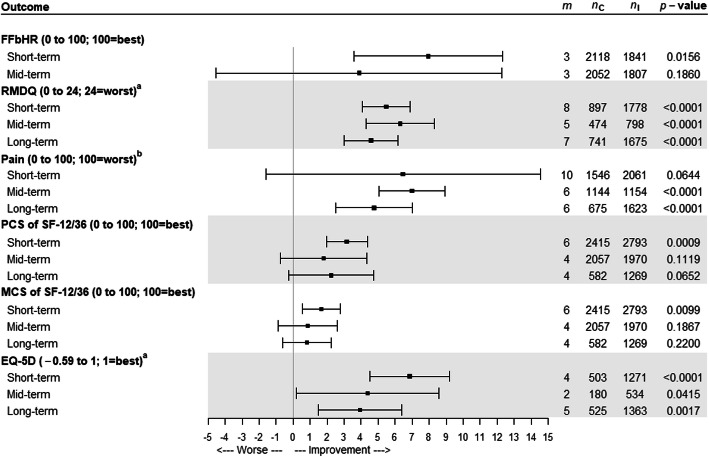
One-step meta-analysis: Estimated difference between control (non-active usual care and sham) and all intervention treatments for each outcome with its 95% confidence intervals adjusted by its baseline value for short-, mid-, and long-term follow-up. Abbreviations: m, number of trials; nC, number of participants in the control arm; nI, number of participants in the intervention arm; short-, mid- and long-term follow-up, measurements taken 2 and 3 months, at 6 months and 12 months post randomisation or entry to the trial, respectively; FFbHR, Hannover functional ability questionnaire for measuring back-pain related functional limitations; RMDQ, Roland Morris disability questionnaire; PCS, physical component scale of SF-12/36; MCS, mental component scale of SF-12/36. **a** The original scale was rescaled from 0 to 100 for graphical representation purposes only. In order to obtain the estimated difference and its 95% confidence interval in its original scale, the value from graph is multiplied by (maximum value/100). For example, the estimated difference for RMDQ at short-term follow-up was 5.47*24/100 = 1.31. **b** One of the following instruments from each trial, where available, was chosen (in descending order): 1. individual VAS on average pain today. 2. average pain over the past 1 week. 3. average pain over the past 2 weeks, average pain over the past 1 month 4. average pain over the past 3 months. 5. the individual item of the CPG pain intensity score (CPG-PS) that is equivalent to the VAS if it is available. 6. the summary score of the CPG-PS or 7. the bodily pain domain of SF-12/36

In our overall one-step meta-analysis (MA) intervention was better than control in improving most outcomes in the short-term (Fig. 1 & Supplementary Table 1). As treatment effects at mid and long-term were generally not statistically significant, we only explored potential moderators for short-term follow-up.

### Identification of moderators

**Fig. 2:**
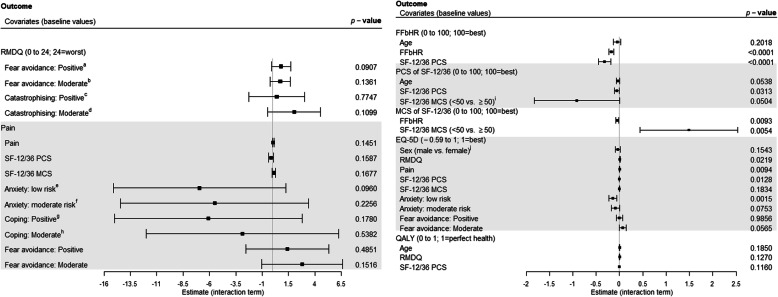
Moderator analysis for short-term outcomes (change from baseline to short-term follow-up) between control (non-active usual care and sham) and all intervention treatments with estimated interaction term and its 95% confidence interval. Abbreviations: RMDQ, Roland Morris disability questionnaire; FFbHR, Hannover functional ability questionnaire for measuring back-pain related functional limitations; QALY, quality-adjusted life-years. **a** estimate of the treatment effect for participants with positive belief (low fear avoidance) of fear avoidance belief was greater as opposed to those with the negative attitude; **b** estimate of the treatment effect for participants with moderate belief of fear avoidance was greater as opposed to those with the negative attitude; **c** estimate of the treatment effect for participants with positive attitude of catastrophising (low catastrophising score) was greater as opposed to those with the negative attitude (high catastrophising score); **d** estimate of the treatment effect for participants with moderate attitude of catastrophising was greater as opposed to those with the negative attitude; **e** estimate of the treatment effect for participants with low risk of anxiety was less as opposed to those with the high risk; **f** estimate of the treatment effect for participants with moderate risk of anxiety was less as opposed to those with the high risk; **g** estimate of the treatment effect for participants with positive attitude of coping strategy (high coping score) was less as opposed to those with the negative attitude (low coping score); **h** estimate of the treatment effect for participants with moderate attitude of coping strategy was less as opposed to those with the negative attitude; **i** estimate of the treatment effect for participants with SF-12/36 MCS score lower than general norm (< 50) was less as opposed to those with score at or above the general norm (≥50); **j** estimate of the treatment effect for male was less as opposed to female

We included potential effect moderators identified from our systematic review [10] and one-step MA in the mixed effects model. In our overall short-term analysis, we found few potential moderator effects (Fig. 2 & Supplementary Table 2). Overall, the baseline value of a measure moderated treatment effects on that measure; FFbHR at baseline moderates the effect on FFbHR, physical component scale (PCS) at baseline moderates the effect on SF-12/36 PCS, and mental component scale (MCS) at baseline moderates the effect on SF-12/36 MCS. Age, gender, LBP disability and severity (FFbHR, RMDQ, Pain and PCS), psychological state (MCS, anxiety, catastrophising and coping) were at least weakly significant in one or more moderator analysis and were considered for further subgroup analysis.

### Recursive partitioning: overall comparison

Analyses included between 1339 and 5208 people (from two to seven trials; Additional file 1: Appendix 2). We identified subgroups for three of the short-term outcome measures; FFbHR, SF-12/36 MCS and SF-12/36 PCS.

**Fig. 3:**
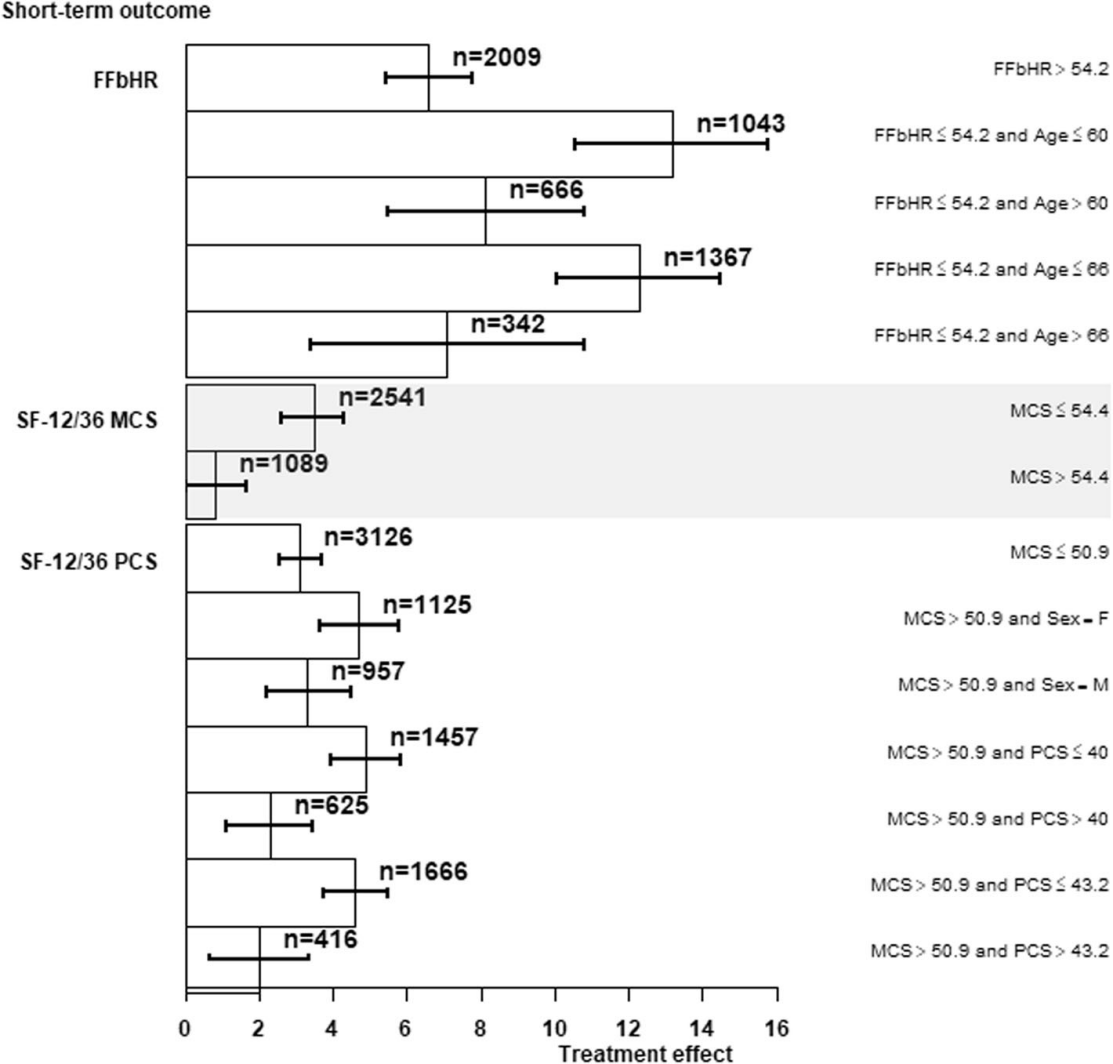
Treatment effect and its 95% confidence interval for each subgroup identified by the RP method for the short-term FFbHR, SF-12/36 MCS and SF-12/36 PCS outcomes

Those with more back pain disability at baseline (FFbHR≤54.2) benefitted more from any therapist-delivered intervention at short-term follow-up than those with FFbHR> 54.2 with treatment effects of 11.3 (95% confidence interval, CI, 9.38 to 13.23) and 6.6 (95% CI, 5.46 to 7.78) respectively, when measured by the FFbHR (Fig. 3). However, those with greater back pain disability (FFbHR≤54.2) and younger (age ≤ 60) gained the greatest benefit on the FFbHR outcome at short-term, with a treatment effect of 13.2 (95% CI, 10.56 to 15.77) compared to those with FFbHR≤54.2 and age > 60, for whom the treatment effect was 8.1 (95% CI, 5.47 to 10.80) (Fig. 3). For the short-term SF-12/36 MCS outcome, those with greater baseline psychological distress gained most benefit (3.5; 95% CI, 2.62 to 4.30) (Fig. 3) from any therapist-delivered intervention. For the short-term SF-12/36 PCS outcome, females with less psychological distress (MCS > 50.9) gained most benefit (4.7; 95% CI, 3.67 to 5.78) or those with less psychological distress (MCS > 50.9) and worse physical disability (PCS ≤ 40) gained more benefit from any therapist-delivered intervention (4.9; 95% CI, 3.96 to 5.82) (Fig. 3).

### Recursive partitioning: pairwise comparisons

Analyses included between 496 and 3879 people (from two to seven trials; Additional file 1: Appendix 3).

#### Active physical vs non-active usual care

No subgroups were identified for the active physical vs non-active usual care comparison.

#### Passive physical vs non-active usual care (Additional file 1: Appendix 4)

For the passive physical vs non-active usual care comparison for short-term FFbHR, those with more back pain disability (FFbHR≤54.2) and younger (age ≤ 53) gained most benefit from passive physical treatments (16.7; 95% CI, 13.16 to 20.18). For the SF-12/36 MCS outcome, those with greater psychological distress (MCS ≤ 54.3) and greater physical disability (PCS ≤ 43.9) gained most benefit (4.3; 95% CI, 3.39 to 5.15).

We found nine subgroups for the PCS outcome when comparing passive physical vs usual care. These can be classified into three subgroups; those with greater physical disability and younger, those with greater physical disability but less psychological distress, and females with greater physical disability but less psychological distress gained most benefit from passive physical treatments.

#### Psychological vs non-active usual care (Additional file 1: Appendix 5)

For the psychological vs non-active usual care comparisons, those with worse disability at baseline (RMDQ> 4) gained most benefit from psychological treatment (1.7; 95% CI, 1.12 to 2.31) for the short-term disability (RMDQ) outcome.

#### Sham control vs non-active usual care (Additional file 1: Appendix 6)

For the sham control vs non-active usual care comparisons, those who were younger (age ≤ 65) or had greater physical disability (PCS ≤ 42) gained most benefit (3.4; 95% CI, 1.80 to 5.04) and (3.1; 95% CI, 1.55 to 4.65), respectively, from sham control on the SF-12/36 MCS outcome.

### Adaptive refinement by directed peeling: overall comparison

Analyses included between 1365 and 5208 people (from two to eight trials; Additional file 1: Appendix 2).

Categorical covariates such as gender and psychological states with three categories (anxiety, catastrophising and coping) were excluded from subgroup identification with ARDP method because a split on these categorical covariates would lead to a large proportion of participants being removed. Additional file 1: Appendix 7 shows the trajectory plot for the interaction treatment effect against the size of the subgroup for short-term (a) FFbHR, (b) RMDQ, (c) Pain, (d) PCS of SF-12/36, (e) MCS of SF-12/36, and (f) EQ-5D. Treatment effects generally increased as subpopulations get smaller but the strong fluctuations for RMDQ (Additional file 1: Appendix 7, figure (b)), Pain (Additional file 1: Appendix 7, figure (c)) and PCS (Additional file 1: Appendix 7, figure (d)) suggest that no subgroup would gain greater improvement in these outcomes.

Table 3 shows the thresholds for selected sizes of the subgroup for the short-term FFbHR found in Additional file 1: Appendix 7, figure (a). The average treatment effect on the short-term FFbHR of approximately 90% of the population (PCS < 48 and MCS < 72) was 8.5. The average treatment effect increased by 8 units to 16.8 in a subpopulation with FFbHR < 29, PCS < 68 and MCS < 57. However, the proportion of participants with such great improvement is very small (approximately 10%). Similarly, 10% of the population (PCS < 29 and MCS < 51) had a very large average treatment effect on the short-term SF-12/36 MCS compared to 90% of the population (PCS < 48 and MCS < 72); 6.0 units compare to 2.2 units (Additional file 1: Appendix 8), suggesting that participants with more psychological distress would gain greater improvement. It is interesting that in the construction of subgroups, the disability scale, FFbHR, did not seem to be an important covariate whereas the functional scale of the SF-12/36 PCS suggested that those with poor physical status would gain greater improvement. Population with low PCS and high RMDQ at baseline (corresponding to poor disability and physical status) also had greater improvement on short-term health utility measured by EQ-5D (Additional file 1: Appendix 9).

**Table 3:** Thresholds for selected sizes of the subgroup for the short-term FFbHR outcome as seen in Additional file 1: Appendix 8, figure (a)

Subgroup size	Age (<)	FFbHR (<) (0 to 100; 100 = best)	SF-12/36 PCS (<) (0 to 100; 100 = best)	SF-12/36 MCS (<) (0 to 100; 100 = best)	Treatment effect
0.001	37	29.17	33.62	28.93	91.29
0.102	91	29.17	67.75	56.82	16.79
0.200	54	54.17	43.62	72.11	13.74
0.308	62	54.17	67.75	72.11	12.72
0.400	62	70.83	40.45	60.35	11.32
0.509	67	62.50	67.75	72.11	10.36
0.602	91	75.00	40.45	60.35	9.96
0.702	91	75.00	47.59	60.35	9.14
0.808	91	100.00	47.59	60.35	8.59
0.906	91	100.00	47.59	72.11	8.47

Adapted with permission from: Patel S, Hee SW, Mistry D, Jordan J, Brown S, Dritsaki M, Ellard DR, Friede T, Lamb SE, Lord J, Madan J, Morris T, Stallard N, Tysall C, Willis A, Underwood M, the Repository G. Programme Grants for Applied Research. Identifying back pain subgroups: developing and applying approaches using individual patient data collected within clinical trials. Southampton (UK): NIHR Journals Library

### Adaptive refinement by directed peeling: pairwise comparisons

#### Active physical vs non-active usual care

In this pairwise comparison, subgroup identification was done for the short-termRMDQ outcome (Additional file 1: Appendix 3). The ARDP method failed to identify subgroups that would gain greater improvement in treatment effect.

#### Passive physical vs non-active usual care

This direct pairwise comparison included FFbHR, SF-12/36 PCS and SF-12/36 MCS outcomes. Similar to the overall analysis, there was no evidence of any subgroup gaining greater treatment effect on the short-term SF-12/36 PCS. Younger participants (< 55 years) with FFbHR < 42 had the greatest treatment effect, 18.42, on the short-term FFbHR. Younger participants (< 51 years) with PCS < 44 (greater physical disability) and MCS < 38 (greater psychological distress) benefited more in short-term SF-12/36 MCS (treatment effect 6.33) when given passive physical treatment compare to non-active usual care (result not shown).

#### Psychological vs non-active usual care

The direct pairwise comparison between psychological and non-active usual care included only RMDQ, finding no subgroup that would gain much treatment effect.

#### Sham control vs non-active usual care

Two trials had sham intervention (sham acupuncture) and collected FFbHR and SF-12/36. There was no treatment effect in different subgroups for the short-term SF-12/36 PCS. Younger, poorer disability and physical limitation, and more psychological distress (< 52 years, FFbHR < 42, PCS < 45 and MCS < 52) participants had greater treatment effect, 12.64, on the short-term FFbHR. Similarly, they (age < 43, PCS < 37 and MCS < 52) had greater treatment effect, 7.86, on the short-term SF-12/36 MCS, suggesting we may be able to identify subgroups responding to sham treatments compared to no treatment (result not shown).

## Discussion

Current LBP treatments offer small to moderate average effects [35], there is therefore, a desire to identify subgroups, targeting patients to treatments most likely to be beneficial.

We have used two statistical methods to identify subgroups defined by participant’s presenting characteristics where treatment effects vary in clinically meaningful ways. In our overall comparison of all interventions with control groups we found that females with low levels of psychological distress gain the greatest benefit on the SF12/36 physical component score from any intervention compared to other participants. This provided proof of principle for our novel methods in this dataset.

It is, however, the pairwise comparisons that are of clinical importance We found the greatest benefit in back pain disability from passive physical treatments (acupuncture or manual therapy) is amongst those that are young, with high levels of disability but low levels of psychological distress.

It is, however, difficult to draw any concrete clinical conclusions with regards to targeting treatments as the effect sizes observed are unlikely to be clinically meaningful and even the small effects sizes seen in the groups that have done less well would still make the intervention useful.

### Other research

Since we started this work, an RCT testing the STarT Back Screening Tool for risk stratification, which had a positive result and was published and included in NICE guidance [36]. This compared standard care to a risk stratification tool that allocated participants to one of three treatment packages delivered by specially trained physiotherapists. The content of the physiotherapy and differences between intervention and control physiotherapists may have contributed to the effect size. The treatment effect moderation of the STarT Back tool was not tested. This trial, therefore, does not materially affect our conclusions.

Further developments in risk stratification tools continue despite challenges of accuracy and application reported by therapists [37]. Some argue for a more multidimensional stratification approach, although our results have not consistently supported this [38]. There are other approaches that might be used to explore these data to identify how participant characteristics might moderate response to different treatments approaches. This is beyond the scope of this current piece of work.

In 2019, after we had completed our work, an IPD meta-analysis of exercise therapy for LBP was published [7]. This work included data from 3514 people from 27 trials. The focus was on exercise interventions only, limiting analysis to moderation effects of single variables, and the inclusion of larger numbers of smaller trials (average size 130) makes this work distinctly different from the work presented here. The authors found some exploratory evidence that those with less physically demanding jobs, or who use pain medication are more likely to benefit from exercise therapy than other treatments in the short term. Lower BMI was also reported to improve outcomes from exercise. In our work, we have focused on therapist-delivered interventions more broadly including active physical, passive physical and psychological treatments rather than just exercise therapy. This has allowed us to include some large high quality trials giving us a much larger overall dataset. The challenge of small low quality studies being included remains.

A 2020 IPD meta-analysis of acupuncture for chronic pain included data from 20,827 people from 39 trials and did not find a subgroup responding exceptionally better to acupuncture [39]. Similarly, an IPD meta-analysis of spinal manipulative therapy (SMT) for chronic low back pain did not find a subgroup that would gain greater benefit from SMT compared to other treatments [40].

#### Strengths

Our large pooled repository with 9328 participants, unlike many previous studies, provides sufficient statistical power for subgroup analyses and may allow future questions in LBP to be addressed without large trials.

We have developed detailed and robust methods for programming and coding of trials, which has been vital in allowing the standardising, coding and pooling of trials that have all come from varied and complex data sets using different coding structures.

As both identified subgroups with very small interactions effects, we feel confident that the statistical methods are robust. We would be more concerned if the methods reported substantially different findings.

### Limitations

Despite our large initial dataset, many analyses used only a small subset of the data because we were unable to pool outcomes measuring the same domain to a common scale [12]. We are confident, however, that the same domain is being measured in each trial.

We did not do a risk of bias assessment for included studies. This would have been important for a review reporting overall treatment effects; and appropriate tools are available. However, for an IPD meta-analysis of this nature exploring sub-group effects we are not aware of any tool to assess risk of bias specifically in moderation effects.

The decision to group trials into active physical, passive physical, psychological, sham and control could be questioned but was necessary for meaningful analyses. Our approach was very carefully considered and agreed by the research and lay team.

For our analyses, we used the mental and physical component scores of the SF-12/36 rather than their eight domain scores. This because we considered these were more clinically relevant as outcomes and to avoid further complicating our analyses, and their interpretation, by adding additional variables. We cannot exclude that an analysis using the individual domain scores as explanatory variables rather than the component scores might have produced a different outcome.

## Conclusion

A large pooled database provided good statistical power for our analyses. In a pooled analysis of any treatment against usual care at baseline, pain, disability, age, gender, and psychological state all showed at least weak evidence of effect moderation on some outcomes. We separated our data into three broad treatment types; active physical, passive physical, and psychological for sub-group analyses. No sub-groups were identified who would benefit more from active physical treatments. Passive physical treatments were most likely to help people who were younger with higher levels of disability and low levels of psychological distress. Psychological treatments were more likely to help those with severe disability. Despite this, the clinical importance of identifying these subgroups is limited. The sizes of sub-groups more likely to benefit and the additional effect sizes observed are small. Positive treatment effects are also seen in groups less likely to benefit. Our analysis indicates no evidence to support the use of sub-grouping to inform treatment choices for people with low back pain. Our methodological approaches worked well and may have applicability in other clinical areas.

## Supplementary Information

**Additional file 1: Appendix 1.** Clinical characteristics at baseline by treatment arms. Data are m, number of trials, and n, number of participants. **Appendix 2.** Summary of the number of trials (*m*), number of participants (*n*) and covariates used to identify subgroup in overall comparison for each short-term outcome with recursive partitioning (RP) and adaptive refinement by directed peeling (ARDP) methods. **Appendix 3.** Summary of the number of trials (*m*), number of participants (*n*) and covariates used to identify subgroup for different pairwise comparisons for each short-term outcome with recursive partitioning (RP) and adaptive refinement by directed peeling (ARDP) methods. **Appendix 4.** Subgroups identified by the recursive partitioning (RP) method for the passive physical vs non-active usual care comparison. **Appendix 5.** Subgroups identified by the recursive partitioning (RP) method for the psychological vs non-active usual care comparison. **Appendix 6.** Subgroups identified by the recursive partitioning (RP) method for the sham vs non-active usual care comparison. **Appendix 7.** Trajectory plot for the treatment effect against the size of the constructed region for short-term (a) Hannover functional ability questionnaire for measuring back-pain related functional limitations, FFbHR, (b) Roland Morris disability questionnaire, RMDQ, (c) Pain, (d) physical component scale of SF-12/36, (e) mental component scale of SF-12/36, and (f) EQ-5D. The number of trials, *m*, and number of patients, *n*, in each of the subgroup identification analyses. **Appendix 8.** Thresholds for selected sizes of the subgroup for the short-term SF-12/36 MCS as seen in Additional file 1: Appendix 7, figure (e). **Appendix 9.** Thresholds for selected sizes of the subgroup for the short-term EQ-5D as seen in Additional file 1: Appendix 7, figure (f).

**Additional file 2: Table S1.** One-step meta-analysis: Estimated difference between control (non-active usual care and sham) and all intervention treatments for each outcome adjusted by its baseline value for short-, mid-, and long-term follow-up.

**Additional file 3: Table S2.** Moderator analysis for short-term outcomes (overall comparison).

## Data Availability

The datasets generated and/or analysed during the current study maybe available from the corresponding authors subject to agreement from the original authors of the included trials.

## Abbreviations

LBP: Low back pain
RCT: Randomised controlled trial
PROMs: Patient reported outcome measures
SD: Standard deviation
RP: Recursive Partitioning
ARDP: Adaptive Refinement by Directed Peeling
RMDQ: Roland Morris disability questionnaire
CPG: Chronic Pain Grade
FFbHR: Hannover functional ability questionnaire
PSFS: Patient specific functional scale
PCS: Physical component scale of SF-12/36
MCS: Mental component scale of SF-12/36
QALY: Quality-adjusted life-years

## References

[1] Foster NE, Anema JR, Cherkin D, Chou R, Cohen SP, Gross DP (2018). Prevention and treatment of low back pain: evidence, challenges, and promising directions. Lancet (London, England).

[2] Buchbinder R, van Tulder M, Oberg B, Costa LM, Woolf A, Schoene M (2018). Low back pain: a call for action. Lancet (London, England).

[3] Hartvigsen J, Hancock MJ, Kongsted A, Louw Q, Ferreira ML, Genevay S (2018). What low back pain is and why we need to pay attention. Lancet (London, England).

[4] Pincus T, Miles C, Froud R, Underwood M, Carnes D, Taylor SJ (2011). Methodological criteria for the assessment of moderators in systematic reviews of randomised controlled trials: a consensus study. BMC Med Res Methodol.

[5] Brookes ST, Whitely E, Egger M, Smith GD, Mulheran PA, Peters TJ (2004). Subgroup analyses in randomized trials: risks of subgroup-specific analyses; power and sample size for the interaction test. J Clin Epidemiol.

[6] Mistry D, Patel S, Hee SW, Stallard N, Underwood M (2014). Evaluating the quality of subgroup analyses in randomized controlled trials of therapist-delivered interventions for nonspecific low back pain: a systematic review. Spine (Phila Pa 1976).

[7] Hayden JA, Wilson MN, Stewart S, Cartwright JL, Smith AO, Riley RD, et al. Exercise treatment effect modifiers in persistent low back pain: an individual participant data meta-analysis of 3514 participants from 27 randomised controlled trials. Br J Sports Med. 2019; bjsports-2019-101205. 10.1136/bjsports-2019-101205 31780447

[8] Hee SW, Dritsaki M, Willis A, Underwood M, Patel S (2017). Development of a repository of individual participant data from randomized controlled trials of therapists delivered interventions for low back pain. Eur J Pain (London, England).

[9] Patel S, Hee SW, Mistry D, Jordan J, Brown S, Dritsaki M (2016). Programme Grants for Applied Research. Identifying back pain subgroups: developing and applying approaches using individual patient data collected within clinical trials.

[10] Gurung T, Ellard DR, Mistry D, Patel S, Underwood M (2015). Identifying potential moderators for response to treatment in low back pain: a systematic review. Physiotherapy..

[11] Chou R, Huffman LH (2007). Nonpharmacologic therapies for acute and chronic low back pain: a review of the evidence for an American pain society/American College of Physicians clinical practice guideline. Ann Intern Med.

[12] Morris T, Hee SW, Stallard N, Underwood M, Patel S (2015). Can we convert between outcome measures of disability for chronic low back pain?. Spine (Phila Pa 1976).

[13] Whitehead A (2003). Meta-Analysis of Controlled Clinical Trials.

[14] Mistry D, Stallard N, Underwood M (2018). A recursive partitioning approach for subgroup identification in individual patient data meta-analysis. Stat Med.

[15] LeBlanc M, Moon J, Crowley J (2005). Adaptive risk group refinement. Biometrics..

[16] Witt CM, Jena S, Selim D, Brinkhaus B, Reinhold T, Wruck K (2006). Pragmatic randomized trial evaluating the clinical and economic effectiveness of acupuncture for chronic low back pain. Am J Epidemiol.

[17] United Kingdom back pain exercise and manipulation (UK BEAM) randomised trial: effectiveness of physical treatments for back pain in primary care. BMJ. 2004;329(7479):1377. 10.1136/bmj.38282.669225.AE PMC535454 15556955

[18] Haake M, Muller HH, Schade-Brittinger C, Basler HD, Schafer H, Maier C (2007). German acupuncture trials (GERAC) for chronic low back pain: randomized, multicenter, blinded, parallel-group trial with 3 groups. Arch Intern Med.

[19] Lamb SE, Hansen Z, Lall R, Castelnuovo E, Withers EJ, Nichols V (2010). Group cognitive behavioural treatment for low-back pain in primary care: a randomised controlled trial and cost-effectiveness analysis. Lancet.

[20] Hay EM, Mullis R, Lewis M, Vohora K, Main CJ, Watson P (2005). Comparison of physical treatments versus a brief pain-management programme for back pain in primary care: a randomised clinical trial in physiotherapy practice. Lancet.

[21] Brinkhaus B, Witt CM, Jena S, Linde K, Streng A, Wagenpfeil S (2006). Acupuncture in patients with chronic low back pain: a randomized controlled trial. Arch Intern Med.

[22] Dufour N, Thamsborg G, Oefeldt A, Lundsgaard C, Stender S (2010). Treatment of chronic low back pain: a randomized, clinical trial comparing group-based multidisciplinary biopsychosocial rehabilitation and intensive individual therapist-assisted back muscle strengthening exercises. Spine (Phila Pa 1976).

[23] Pengel LH, Refshauge KM, Maher CG, Nicholas MK, Herbert RD, McNair P (2007). Physiotherapist-directed exercise, advice, or both for subacute low back pain: a randomized trial. Ann Intern Med.

[24] Thomas KJ, MacPherson H, Thorpe L, Brazier J, Fitter M, Campbell MJ (2006). Randomised controlled trial of a short course of traditional acupuncture compared with usual care for persistent non-specific low back pain. BMJ.

[25] Hancock MJ, Maher CG, Latimer J, McLachlan AJ, Cooper CW, Day RO (2007). Assessment of diclofenac or spinal manipulative therapy, or both, in addition to recommended first-line treatment for acute low back pain: a randomised controlled trial. Lancet.

[26] Von Korff M, Balderson BH, Saunders K, Miglioretti DL, Lin EH, Berry S (2005). A trial of an activating intervention for chronic back pain in primary care and physical therapy settings. Pain..

[27] Carr JL, Klaber Moffett JA, Howarth E, Richmond SJ, Torgerson DJ, Jackson DA (2005). A randomized trial comparing a group exercise programme for back pain patients with individual physiotherapy in a severely deprived area. Disabil Rehabil.

[28] Moore JE, Von Korff M, Cherkin D, Saunders K, Lorig K (2000). A randomized trial of a cognitive-behavioral program for enhancing back pain self care in a primary care setting. Pain..

[29] Smeets RJ, Vlaeyen JW, Hidding A, Kester AD, van der Heijden GJ, van Geel AC (2006). Active rehabilitation for chronic low back pain: cognitive-behavioral, physical, or both? First direct post-treatment results from a randomized controlled trial [ISRCTN22714229]. BMC Musculoskelet Disord.

[30] Cecchi F, Molino-Lova R, Chiti M, Pasquini G, Paperini A, Conti AA (2010). Spinal manipulation compared with back school and with individually delivered physiotherapy for the treatment of chronic low back pain: a randomized trial with one-year follow-up. Clin Rehabil.

[31] Moffett JK, Torgerson D, Bell-Syer S, Jackson D, Llewlyn-Phillips H, Farrin A (1999). Randomised controlled trial of exercise for low back pain: clinical outcomes, costs, and preferences. BMJ.

[32] Macedo LG, Latimer J, Maher CG, Hodges PW, McAuley JH, Nicholas MK (2012). Effect of motor control exercises versus graded activity in patients with chronic nonspecific low back pain: a randomized controlled trial. Phys Ther.

[33] Carlsson CP, Sjolund BH (2001). Acupuncture for chronic low back pain: a randomized placebo-controlled study with long-term follow-up. Clin J Pain.

[34] Kennedy S, Baxter GD, Kerr DP, Bradbury I, Park J, McDonough SM (2008). Acupuncture for acute non-specific low back pain: a pilot randomised non-penetrating sham controlled trial. Complement Ther Med.

[35] Keller A, Hayden J, Bombardier C, van Tulder M (2007). Effect sizes of non-surgical treatments of non-specific low-back pain. Eur Spine J.

[36] Hill JC, Whitehurst DG, Lewis M, Bryan S, Dunn KM, Foster NE (2011). Comparison of stratified primary care management for low back pain with current best practice (STarT Back): a randomised controlled trial. Lancet.

[37] Brunner E, Dankaerts W, Meichtry A, O'Sullivan K, Probst M (2018). Physical Therapists' ability to identify psychological factors and their self-reported competence to manage chronic low Back pain. Phys Ther.

[38] Rampersaud YR, Bidos A, Fanti C, Perruccio AV (2017). The need for multidimensional stratification of chronic low Back pain (LBP). Spine (Phila Pa 1976).

[39] Foster NE, Vertosick EA, Lewith G, Linde K, MacPherson H, Sherman KJ (2020). Identifying patients with chronic pain who respond to acupuncture: results from an individual patient data meta-analysis. Acupunct Med.

[40] de Zoete A, de Boer MR, Rubinstein SM, van Tulder MW, Underwood M, Hayden JA, et al. Moderators of the effect of spinal manipulative therapy on pain relief and function in patients with chronic low back pain: An individual participant data meta-analysis. Spine (Phila Pa 1976). 2020. 10.1097/BRS.0000000000003814 PMC7993913 33186277

