# Supplementary material for: Identification of subgroup effect with an individual participant data meta-analysis of randomised controlled trials of three different types of therapist-delivered care in low back pain
Source: BMC Musculoskelet Disord. 2021 Feb 16;22:191. doi: 10.1186/s12891-021-04028-8 (PMC7885433; doi:10.1186/s12891-021-04028-8)
Supplement: Supplementary file 1 — Additional file 1: Appendix 1. Clinical characteristics at baseline by treatment arms. Data are m, number of trials, and n, number of participants. Appendix 2. Summary of the number of trials (m), number of participants (n) and covariates used to identify subgroup in overall comparison for each short-term outcome with recursive partitioning (RP) and adaptive refinement by directed peeling (ARDP) methods. Appendix 3. Summary of the number of trials (m), number of participants (n) and covariates used to identify subgroup for different pairwise comparisons for each short-term outcome with recursive partitioning (RP) and adaptive refinement by directed peeling (ARDP) methods. Appendix 4. Subgroups identified by the recursive partitioning (RP) method for the passive physical vs non-active usual care comparison. Appendix 5. Subgroups identified by the recursive partitioning (RP) method for the psychological vs non-active usual care comparison. Appendix 6. Subgroups identified by the recursive partitioning (RP) method for the sham vs non-active usual care comparison. Appendix 7. Trajectory plot for the treatment effect against the size of the constructed region for short-term (a) Hannover functional ability questionnaire for measuring back-pain related functional limitations, FFbHR, (b) Roland Morris disability questionnaire, RMDQ, (c) Pain, (d) physical component scale of SF-12/36, (e) mental component scale of SF-12/36, and (f) EQ-5D. The number of trials, m, and number of patients, n, in each of the subgroup identification analyses. Appendix 8. Thresholds for selected sizes of the subgroup for the short-term SF-12/36 MCS as seen in Additional file 1: Appendix 7, figure (e). Appendix 9. Thresholds for selected sizes of the subgroup for the short-term EQ-5D as seen in Additional file 1: Appendix 7, figure (f). [file 12891_2021_4028_MOESM1_ESM.docx]

**Appendix 1: Clinical characteristics at baseline by treatment arms. Data are m, number of trials, and n, number of participants.**

| **Characteristics** | | **Control^a^** | | | **Intervention^b^** | | | **All** | |
| --- | --- | --- | --- | --- | --- | --- | --- | --- | --- |
|  |  | **No. of trials *m*=14** | | **No. of participants *n*=3573** | **No. of trials *m*=19** | **No. of participants *n*=5755** | | **No. of trials *m*=19** | **No. of participants *n*=9328** |
| ***Physical disability*** | |  | |  |  |  | |  |  |
| *CPG-DS (0 to 100; 100=worst)^a^* | |  | |  |  |  | |  |  |
|  | No. of trials, *m*: no. of participants, *n* | 4 | | 1439 | 4 | 1889 | | 4 | 3328 |
|  | Mean (SD) | 51.35 | | (21.46) | 49.26 | (22.35) | | 50.16 | (21.99) |
| *FFbHR (0 to 100; 100=best)* | |  | |  |  |  | |  |  |
|  | No. of trials, *m*: no. of participants, *n* | 3 | | 2249 | 3 | 1927 | | 3 | 4176 |
|  | Mean (SD) | 57.06 | | (20.37) | 58.33 | (20.63) | | 57.64 | (20.5) |
| *ODI (0 to 100; 100=worst)* | |  | |  |  |  | |  |  |
|  | No. of trials, *m*: no. of participants, *n* | 1 | | 80 | 1 | 159 | | 1 | 239 |
|  | Mean (SD) | 31.36 | | (14.24) | 33.72 | (15.40) | | 32.93 | (15.03) |
| *PDI (0 to 70; 70=worst)* | |  | |  |  |  | |  |  |
|  | No. of trials, *m*: no. of participants, *n* | 1 | | 152 | 1 | 146 | | 1 | 298 |
|  | Mean (SD) | 31.23 | | (12.26) | 28.92 | (11.12) | | 30.10 | (11.75) |
| *PSFS (0 to 10; 10=best)* | |  | |  |  |  | |  |  |
|  | No. of trials, *m*: no. of participants, *n* | 2 | | 188 | 3 | 479 | | 3 | 667 |
|  | Mean (SD) | 3.97 | | (1.84) | 3.71 | (1.73) | | 3.79 | (1.76) |
| *RMDQ (0 to 24; 24=worst)* | |  | |  |  |  | |  |  |
|  | No. of trials, *m*: no. of participants, *n* | 9 | | 1150 | 14 | 3560 | | 14 | 4710 |
|  | Mean (SD) | 9.03 | | (5.04) | 10.19 | (5.08) | | 9.91 | (5.09) |
| *Troublesomeness* | |  | |  |  |  | |  |  |
|  | No. of trials, *m* | 3 | |  | 4 |  | | 4 |  |
|  | Not at all troublesome (%) | 4 | | (0.66) | 7 | (0.43) | | 11 | (0.49) |
|  | Slightly troublesome (%) | 51 | | (8.44) | 158 | (9.65) | | 209 | (9.32) |
|  | Moderately troublesome (%) | 284 | | (47.02) | 724 | (44.20) | | 1008 | (44.96) |
|  | Very troublesome (%) | 211 | | (34.93) | 578 | (35.29) | | 789 | (35.19) |
|  | Extremely troublesome (%) | 54 | | (8.94) | 171 | (10.44) | | 225 | (10.04) |
| ***Pain*** | |  | |  |  |  | |  |  |
| *CPG-PS (0 to 100; 100=worst)^c^* | |  | |  |  |  | |  |  |
|  | No. of trials, *m*: no. of participants, *n* | 4 | | 1441 | 4 | 1898 | | 5 | 3339 |
|  | Mean (SD) | 63.98 | | (16.52) | 61.66 | (17.83) | | 62.66 | (17.31) |
| *Average pain today (0 to 100; 100=worst)* | | | | | | | | | |
|  | No. of trials, *m*: no. of participants, *n* | 2 | | 171 | 4 | 971 | | 4 | 1142 |
|  | Mean (SD) | 59.37 | | (20.92) | 55.02 | (23.15) | | 55.67 | (22.87) |
| *Average pain over the past 1 week (0 to 100; 100=worst)* | | | | | | | | | |
|  | No. of trials, *m*: no. of participants, *n* | 2 | | 84 | 4 | 797 | | 4 | 881 |
|  | Mean (SD) | 52.69 | | (18.23) | 63.25 | (21.51) | | 62.24 | (21.44) |
| *Average pain over the past 2 weeks (0 to 100; 100=worst)* | | | | | | | | | |
|  | No. of trials, *m*: no. of participants, *n* | 0 | | 0 | 1 | 286 | | 1 | 286 |
|  | Mean (SD) | - | | - | 58.02 | (19.42) | | 58.02 | (19.42) |
| *Average pain over the past 4 weeks (0 to 100; 100=worst)* | | | | | | | | | |
|  | No. of trials, *m*: no. of participants, *n* | 1 | | 24 | 1 | 24 | | 1 | 48 |
|  | Mean (SD) | 62.58 | | (19.72) | 56.17 | (27.67) | | 59.38 | (23.99) |
| *Worst pain today (0 to 100; 100=worst)* | | | | | | | | | |
|  | No. of trials, *m*: no. of participants, *n* | 1 | | 51 | 1 | 172 | | 1 | 223 |
|  | Mean (SD) | 82.16 | | (17.47) | 81.43 | (18.06) | | 81.60 | (17.89) |
| *Worst pain over the past 2 weeks (0 to 100; 100=worst)* | | | | | | | | | |
|  | No. of trials, *m*: no. of participants, *n* | 0 | | 0 | 1 | 286 | | 1 | 286 |
|  | Mean (SD) | - | | - | 74.74 | (19.59) | | 74.74 | (19.59) |
| *Worst pain over the past 4 weeks (0 to 100; 100=worst)* | | | | | | | | | |
|  | No. of trials, *m*: no. of participants, *n* | 1 | | 24 | 1 | 24 | | 1 | 48 |
|  | Mean (SD) | 73.79 | | (20.04) | 76.42 | (22.69) | | 75.10 | (21.22) |
| ***Quality of life*** | | | | | | | | | |
| *SF-12/36 PCS (0 to 100; 100=best)* | | | | | | | | | |
|  | No. of trials, *m*: no. of participants, *n* | 6 | | 2722 | 9 | 3973 | | 9 | 6695 |
|  | Mean (SD) | 35.72 | | (8.58) | 36.51 | (8.07) | | 36.19 | (8.29) |
| *SF-12/36 MCS (0 to 100; 100=best)* | | | | | | | | | |
|  | No. of trials, *m*: no. of participants, *n* | 6 | 2722 | | 9 | | 3973 | 9 | 6695 |
|  | Mean (SD) | 46.01 | (11.82) | | 44.67 | | (11.92) | 45.22 | (11.90) |
| ***Health utility*** | |  |  | |  | |  |  |  |
| *EQ-5D-3L (-0.11 to 1;1=best)* | |  |  | |  | |  |  |  |
|  | No. of trials, *m*: no. of participants, *n* | 5 | 724 | | 7 | | 2356 | 7 | 3080 |
|  | Mean (SD) | 0.59 | (0.26) | | 0.59 | | (0.27) | 0.59 | (0.27) |
| ***Depression*** | |  |  | |  | |  |  |  |
| *DASS-DE (0 to 42; 42=worst)* | |  |  | |  | |  |  |  |
|  | No. of trials, *m*: no. of participants, *n* | 1 | 68 | | 1 | | 190 | 1 | 258 |
|  | Mean (SD) | 7.06 | (7.61) | | 7.24 | | (8.07) | 7.19 | (7.94) |
| *DRAM* | |  |  | |  | |  |  |  |
|  | No. of trials, *m* | 2 |  | | 2 | |  | 2 |  |
|  | Type N (%) | 184 | (44.88) | | 373 | | (36.89) | 557 | (39.2) |
|  | Type R (%) | 158 | (38.54) | | 414 | | (40.95) | 572 | (40.25) |
|  | Type DD (%) | 49 | (11.95) | | 142 | | (14.05) | 191 | (13.44) |
|  | Type DS (%) | 19 | (4.63) | | 82 | | (8.11) | 101 | (7.11) |
| *HADS-DE (0 to 21; 21=worst)* | |  |  | |  | |  |  |  |
|  | No. of trials, *m*: no. of participants, *n* | 1 | 231 | | 1 | | 464 | 1 | 695 |
|  | Mean (SD) | 5.54 | (3.60) | | 6.04 | | (3.81) | 5.87 | (3.75) |
| *MZDI (0 to 69; 69=worst)* | |  |  | |  | |  |  |  |
|  | No. of trials, *m*: no. of participants, *n* | 2 | 411 | | 3 | | 1313 | 3 | 1724 |
|  | Mean (SD) | 19.77 | (10.75) | | 21.46 | | (10.66) | 21.06 | (10.70) |
| ***Anxiety*** | |  |  | |  | |  |  |  |
| *DASS-AN (0 to 42; 42=worst)* | |  |  | |  | |  |  |  |
|  | No. of trials, *m*: no. of participants, *n* | 1 | 68 | | 1 | | 190 | 1 | 258 |
|  | Mean (SD) | 5.35 | (6.92) | | 5.41 | | (7.23) | 5.40 | (7.14) |
| *HADS-AN (0 to 21; 21=worst)* | |  |  | |  | |  |  |  |
|  | No. of trials, *m*: no. of participants, *n* | 1 | 230 | | 1 | | 458 | 1 | 688 |
|  | Mean (SD) | 7.49 | (4.43) | | 8.22 | | (4.30) | 7.98 | (4.35) |
| ***Fear avoidance*** | |  |  | |  | |  |  |  |
| *ALBPSQ-FA (0 to 30; 30=worst)* | |  |  | |  | |  |  |  |
|  | No. of trials, *m*: no. of participants, *n* | 1 | 33 | | 2 | | 274 | 2 | 307 |
|  | Mean (SD) | 18.42 | (5.90) | | 18.20 | | (6.47) | 18.22 | (6.40) |
| *FABQ-PC (0 to 24; 24=worst)* | |  |  | |  | |  |  |  |
|  | No. of trials, *m*: no. of participants, *n* | 5 | 1522 | | 5 | | 1960 | 5 | 3482 |
|  | Mean (SD) | 16.49 | (5.48) | | 15.33 | | (5.65) | 15.84 | (5.61) |
| *TSK (16 to 68; 68=worst)* | |  |  | |  | |  |  |  |
|  | No. of trials, *m*: no. of participants, *n* | 4 | 353 | | 5 | | 949 | 5 | 1302 |
|  | Mean (SD) | 39.40 | (8.51) | | 41.31 | | (7.91) | 40.79 | (8.12) |
| ***Catastrophising*** | |  |  | |  | |  |  |  |
| *CSQ-CAT (0 to 36; 36=worst)* | |  |  | |  | |  |  |  |
|  | No. of trials, *m*: no. of participants, *n* | 0 | 0 | | 2 | | 561 | 2 | 561 |
|  | Mean (SD) | - | - | | 9.19 | | (7.16) | 9.19 | (7.16) |
| *PRSS-CAT (0 to 45; 45=worst)* | |  |  | |  | |  |  |  |
|  | No. of trials, *m*: no. of participants, *n* | 2 | 188 | | 2 | | 309 | 2 | 497 |
|  | Mean (SD) | 17.23 | (8.53) | | 17.21 | | (8.92) | 17.22 | (8.77) |
| ***Coping*** | |  |  | |  | |  |  |  |
| *CSQ-CSS (0 to 36; 36=best)* | |  |  | |  | |  |  |  |
|  | No. of trials, *m*: no. of participants, *n* | 0 | 0 | | 1 | | 394 | 1 | 394 |
|  | Mean (SD) | - | - | | 25.23 | | (6.43) | 25.23 | (6.43) |
| *PRSS-CSS (0 to 45; 45=best)* | |  |  | |  | |  |  |  |
|  | No. of trials, *m*: no. of participants, *n* | 2 | 188 | | 2 | | 309 | 2 | 497 |
|  | Mean (SD) | 31.97 | (6.85) | | 30.61 | | (7.29) | 31.13 | (7.15) |
| *PSEQ (0 to 60; 60=best)* | |  |  | |  | |  |  |  |
|  | No. of trials, *m*: no. of participants, *n* | 2 | 290 | | 4 | | 1049 | 4 | 1339 |
|  | Mean (SD) | 41.74 | (12.76) | | 40.10 | | (12.93) | 40.46 | (12.90) |
| ***Somatic perception*** | |  |  | |  | |  |  |  |
| *MSPQ (0 to 39; 39=worst)* | |  |  | |  | |  |  |  |
|  | No. of trials, *m*: no. of participants, *n* | 2 | 411 | | 3 | | 1403 | 3 | 1814 |
|  | Mean (SD) | 6.14 | (5.34) | | 6.55 | | (5.31) | 6.45 | (5.32) |
| ***Sensory index*** | |  |  | |  | |  |  |  |
| *McGill-SE (0 to 33; 33=worst)* | |  |  | |  | |  |  |  |
|  | No. of trials, *m*: no. of participants, *n* | 0 | 0 | | 1 | | 355 | 1 | 355 |
|  | Mean (SD) | - | - | | 14.24 | | (6.22) | 14.24 | (6.22) |
| *SES-SE (10 to 40; 40=worst)* | |  |  | |  | |  |  |  |
|  | No. of trials, *m*: no. of participants, *n* | 1 | 152 | | 1 | | 146 | 1 | 298 |
|  | Mean (SD) | 49.45 | (9.84) | | 49.70 | | (9.05) | 49.57 | (9.45) |
| ***Affective index*** | |  |  | |  | |  |  |  |
| *McGill-AF (0 to 12; 12=worst)* | |  |  | |  | |  |  |  |
|  | No. of trials, *m*: no. of participants, *n* | 0 | 0 | | 1 | | 379 | 1 | 379 |
|  | Mean (SD) | - | - | | 4.23 | | (3.33) | 4.23 | (3.33) |
| *SES-AF (14 to 56; 56=worst)* | |  |  | |  | |  |  |  |
|  | No. of trials, *m*: no. of participants, *n* | 1 | 152 | | 1 | | 146 | 1 | 298 |
|  | Mean (SD) | 50.43 | (8.78) | | 50.19 | | (8.38) | 50.31 | (8.57) |
| a Control arm includes best usual care and sham treatments. Four trials did not have control arm.  b Intervention arm includes active physical, passive physical, psychological and combination.  c Two trials with 4 weeks recall period and three trials with 3 months recall period.  Abbreviations: BMI, body mass index; CPG-DS, chronic pain grade disability score; FFbHR, Hannover functional ability questionnaire for measuring back-pain related functional limitations; ODI, Oswestry disability index; PDI, pain disability index; PSFS, patient specific functional scale; RMDQ, Roland Morris disability questionnaire; CPG-PS, chronic pain grade pain intensity score; PCS, physical component scale of SF-12/36; MCS, mental component scale of SF-12/36; DASS, depression anxiety stress scales; DRAM, distress and risk assessment method; Type N, normal; Type R, at risk; Type DD, distressed-depressive; Type DS, distressed-somatic; HADS, hospital anxiety and depression scale; MZDI, modified Zung depression index; ALBPSQ, acute low back pain screening questionnaire; FABQ, fear-avoidance beliefs questionnaire; TSK, Tampa scale for kinesiophobia; CSQ, coping strategy questionnaire; PRSS, pain related self-statement; PSEQ, pain self-efficacy questionnaire; MSPQ, modified somatic perception questionnaire; SES, pain experience scale (Schmerzempfindungsskala). | | | | | | | | | |

Adapted with permission from: Patel S, Hee SW, Mistry D, Jordan J, Brown S, Dritsaki M, Ellard DR, Friede T, Lamb SE, Lord J, Madan J, Morris T, Stallard N, Tysall C, Willis A, Underwood M, the Repository G. Programme Grants for Applied Research. Identifying back pain subgroups: developing and applying approaches using individual patient data collected within clinical trials. Southampton (UK): NIHR Journals Library

**Appendix 2: Summary of the number of trials (*m*), number of participants (*n*) and covariates used to identify subgroup in overall comparison for each short-term outcome with recursive partitioning (RP) and adaptive refinement by directed peeling (ARDP) methods.**

|  | **RP** | | | **ARDP** | | |
| --- | --- | --- | --- | --- | --- | --- |
| **Outcomes** | ***m*** | ***n**** | **Covariates** | ***m*** | ***n**** | **Covariates** |
| Pain | 2[17,19] | 1377 | Age, sex, anxiety, fear avoidance, MCS, PCS, pain, RMDQ | 3[17,18,  19] | 2534 | Age, pain, PCS, MCS |
| EQ-5D | 2[17,19] | 1339 | Age, sex, anxiety, fear avoidance, MCS, PCS, pain, RMDQ | 2[17,19] | 1365 | RMDQ, pain, PCS, MCS |
| FFbHR | 3[16,18,21] | 3718 | Age, sex, PCS, FFbHR, MCS | 3[16,18,21] | 3718 | Age, FFbHR, PCS, MCS |
| MCS of SF-12/36 | 3[16,18,21] | 3630 | Age, sex, FFbHR, MCS, PCS | 3[16,18,21] | 3630 | Age, FFbHR, PCS, MCS |
| PCS of SF-12/36 | 6[16,17,18,19,21,24] | 5208 | Age, sex, MCS, PCS | 6[16,17,18,19,21,24] | 5208 | Age, PCS, MCS |
| RMDQ | 7[17,19,23,25,26,28,29] | 2564 | Age, sex, fear avoidance, RMDQ | 8[17,19,23,25,26,28,29,34] | 2675 | Age, RMDQ |
| QALY | 4[17,19,29,31] | 1514 | Age, RMDQ |  |  |  |

FFbHR, Hannover functional ability questionnaire for measuring back-pain related functional limitations; RMDQ, Roland Morris disability questionnaire; PCS, physical component scale of SF-12/36; MCS, mental component scale of SF-12/36; QALY, quality-adjusted life-years.

*Figures vary as RP includes more variables and if the additional variables have missing values then these are dropped, hence why lower figures are observed for RP compared to ARDP.

**Appendix 3: Summary of the number of trials (*m*), number of participants (*n*) and covariates used to identify subgroup for different pairwise comparisons for each short-term outcome with recursive partitioning (RP) and adaptive refinement by directed peeling (ARDP) methods.**

|  |  | **RP** | | | **ARDP** | | |
| --- | --- | --- | --- | --- | --- | --- | --- |
| **Pairwise comparison** | **Outcomes** | ***m*** | ***n**** | **Covariates** | ***m*** | ***n**** | **Covariates** |
| Active physical vs. non-active usual care | RMDQ | 2[17,29] | 576 | Fear avoidance,  age, sex, RMDQ,  pain, EQ-5D, anxiety | 2[17,29] | 622 | Age, RMDQ |
|  | QALY | 2[17,31] | 496 | Age, RMDQ |  |  |  |
| Passive physical vs. non-active usual care | FFbHR | 3[16,18,21] | 3272 | Age, PCS, FFbHR, sex, MCS | 3[16,18,21] | 3272 | Age, FFbHR, PCS, MCS |
|  | PCS | 5[16,17,18,21,24] | 3879 | Age, sex, MCS, PCS | 5[16,17,18,21,24] | 3879 | Age, PCS, MCS |
|  | MCS | 5[16,17,18,21,24] | 3879 | Age, sex, MCS, PCS | 5[16,17,18,21,24] | 3879 | Age, PCS, MCS |
|  | QALY | 3[17,18,24] | 1209 | Age, PCS |  |  |  |
| Psychological vs. non-active usual care | RMDQ | 3[19,26,28] | 928 | Fear avoidance, age, sex, RMDQ, pain | 3[19,26,28] | 957 | Age, RMDQ |
| Sham control vs. non-active usual care | FFbHR | 2[18,21] | 881 | Age, sex, MCS, PCS | 2[18,21] | 881 | Age, FFbHR, PCS, MCS |
|  | PCS | 2[18,21] | 879 | Age, sex, MCS, PCS | 2[18,21] | 879 | Age, PCS, MCS |
|  | MCS | 2[18,210 | 879 | Age, sex, MCS, PCS | 2[18,210 | 879 | Age, PCS, MCS |

FFbHR, Hannover functional ability questionnaire for measuring back-pain related functional limitations; RMDQ, Roland Morris disability questionnaire; PCS, physical component scale of SF-12/36; MCS, mental component scale of SF-12/36; QALY, quality-adjusted life-years.

*Figures vary as RP includes more variables and if the additional variables have missing values then these are dropped, hence why lower figures are observed for RP compared to ARDP.

Appendix 4: Subgroups identified by the recursive partitioning (RP) method for the passive physical vs non-active usual care comparison

| Subgroups | *n* | Treatment effect (95% confidence interval, CI) | Interaction effect | Unadjusted *p*-value |
| --- | --- | --- | --- | --- |
| ***Outcome: short-term FFbHR*** |  |  |  |  |
| ***Overall treatment effect (95% CI)***: 9.95 (8.80, 11.11) |  |  |  |  |
| *Candidate 1* |  |  |  |  |
| FFbHR ≤ 54.2 | 1,424 | 12.9 (10.81, 14.91) | 5.45 | <0.001 |
| FFbHR > 54.2 | 1,848 | 7.4 (6.23, 8.59) |  |  |
|  |  |  |  |  |
| *Candidate 2* |  |  |  |  |
| FFbHR ≤ 54.2 AND Age ≤ 57 | 731 | 15.9 (12.80, 18.92) | 6.63 | 0.002 |
| FFbHR ≤ 54.2 AND Age > 57 | 693 | 9.2 (6.64, 11.82) |  |  |
|  |  |  |  |  |
| *Candidate 3* |  |  |  |  |
| FFbHR ≤ 54.2 AND Age ≤ 53 | 571 | 16.7 (13.16, 20.18) | 6.85 | 0.001 |
| FFbHR ≤ 54.2 AND Age > 53 | 853 | 9.8 (7.43, 12.22) |  |  |
|  |  |  |  |  |
| *Candidate 4* |  |  |  |  |
| FFbHR ≤ 41.7 | 792 | 15.0 (12.06, 18.01) | 6.71 | <0.001 |
| FFbHR > 41.7 | 2,480 | 8.3 (7.19, 9.45) |  |  |
|  |  |  |  |  |
| ***Outcome: short-term MCS*** |  |  |  |  |
| ***Overall treatment effect (95% CI)***: 2.96 (2.31, 3.61) |  |  |  |  |
| *Candidate 1* |  |  |  |  |
| MCS ≤ 54.3 | 2,714 | 3.8 (2.97, 4.55) | 2.82 | <0.001 |
| MCS > 54.3 | 1,165 | 0.9 (0.10, 1.76) |  |  |
|  |  |  |  |  |
| *Candidate 2* |  |  |  |  |
| MCS ≤ 54.3 AND PCS ≤ 43.9 | 2,171 | 4.3 (3.39, 5.15) | 2.43 | 0.019 |
| MCS ≤ 54.3 AND PCS > 43.9 | 543 | 1.9 (0.11, 3.59) |  |  |
|  |  |  |  |  |
| *Candidate 3* |  |  |  |  |
| MCS ≤ 51.3 | 2,327 | 3.8 (2.96, 4.70) | 2.57 | <0.001 |
| MCS > 51.3 | 1,552 | 1.3 (0.52, 1.99) |  |  |
|  |  |  |  |  |
| ***Outcome: short-term PCS*** |  |  |  |  |
| ***Overall treatment effect (95% CI)***: 4.10 (3.56, 4.63) |  |  |  |  |
| *Candidate 1* |  |  |  |  |
| PCS ≤ 43.6 | 3,103 | 4.4 (3.78, 4.99) | 1.61 | 0.013 |
| PCS > 43.6 | 776 | 2.8 (1.87, 3.67) |  |  |
|  |  |  |  |  |
| *Candidate 2* |  |  |  |  |
| PCS ≤ 43.6 AND Age ≤ 44 | 942 | 5.4 (4.21, 6.49) | 1.45 | 0.040 |
| PCS ≤ 43.6 AND Age > 44 | 2,161 | 3.9 (3.20, 4.60) |  |  |
|  |  |  |  |  |
| *Candidate 3* |  |  |  |  |
| PCS ≤ 37.8 | 2,326 | 4.6 (3.90, 5.32) | 1.23 | 0.025 |
| PCS > 37.8 | 1,553 | 3.4 (2.66, 4.09) |  |  |
|  |  |  |  |  |
| *Candidate 4* |  |  |  |  |
| PCS ≤ 37.8 AND Age ≤ 62 | 1,682 | 5.1 (4.21, 5.94) | 1.97 | 0.016 |
| PCS ≤ 37.8 AND Age > 62 | 644 | 3.1 (1.94, 4.28) |  |  |
|  |  |  |  |  |
| *Candidate 5* |  |  |  |  |
| PCS ≤ 37.8 AND MCS > 44.0 | 1,396 | 5.5 (4.55, 6.41) | 1.80 | 0.011 |
| PCS ≤ 37.8 AND MCS ≤ 44.0 | 930 | 3.7 (2.64, 4.71) |  |  |
|  |  |  |  |  |
| *Candidate 6* |  |  |  |  |
| PCS ≤ 37.8 AND MCS > 51.8 | 932 | 5.8 (4.63, 6.91) | 1.78 | 0.012 |
| PCS ≤ 37.8 AND MCS ≤ 51.8 | 1,394 | 4.0 (3.11, 4.87) |  |  |
|  |  |  |  |  |
| *Candidate 7* |  |  |  |  |
| PCS ≤ 37.8 AND MCS > 51.8 AND Sex = Female | 520 | 6.6 (5.12, 8.16) | 1.73 | 0.167 |
| PCS ≤ 37.8 AND MCS > 51.8 AND Sex = Male | 412 | 4.9 (3.17, 6.65) |  |  |
|  |  |  |  |  |
| *Candidate 8* |  |  |  |  |
| PCS ≤ 40.3 | 2,715 | 4.5 (3.85, 5.16) | 1.61 | 0.006 |
| PCS > 40.3 | 1,164 | 2.9 (2.11, 3.68) |  |  |
|  |  |  |  |  |
| *Candidate 9* |  |  |  |  |
| PCS ≤ 40.3 AND MCS > 51.5 | 1,086 | 5.4 (4.37, 6.48) | 1.38 | 0.042 |
| PCS ≤ 40.3 AND MCS ≤ 51.5 | 1,629 | 4.1 (3.24, 4.85) |  |  |
| *Note: The first row of each candidate subgroup is the selected subgroup with enhanced treatment effect.*  FFbHR, Hannover functional ability questionnaire for measuring back-pain related functional limitations; RMDQ, Roland Morris disability questionnaire; PCS, physical component scale of SF-12/36; MCS, mental component scale of SF-12/36. | | | | |

Reproduced with permission from: Patel S, Hee SW, Mistry D, Jordan J, Brown S, Dritsaki M, Ellard DR, Friede T, Lamb SE, Lord J, Madan J, Morris T, Stallard N, Tysall C, Willis A, Underwood M, the Repository G. Programme Grants for Applied Research. Identifying back pain subgroups: developing and applying approaches using individual patient data collected within clinical trials. Southampton (UK): NIHR Journals Library

Appendix 5: Subgroups identified by the recursive partitioning (RP) method for the psychological vs non-active usual care comparison

| Subgroups | *n* | Treatment effect (95% confidence interval, CI) | Interaction effect | Unadjusted *p*-value |
| --- | --- | --- | --- | --- |
| ***Outcome: short-term RMDQ*** |  |  |  |  |
| ***Overall treatment effect (95% CI)***: 1.40 (0.89, 1.91) |  |  |  |  |
| *Candidate 1* |  |  |  |  |
| RMDQ > 4 | 697 | 1.7 (1.12, 2.31) | 1.07 | 0.038 |
| RMDQ ≤ 4 | 231 | 0.7 (-0.11, 1.40) |  |  |
| *Note: The first row of each candidate subgroup is the selected subgroup with enhanced treatment effect.*  RMDQ, Roland Morris disability questionnaire. | | | | |

Reproduced with permission from: Patel S, Hee SW, Mistry D, Jordan J, Brown S, Dritsaki M, Ellard DR, Friede T, Lamb SE, Lord J, Madan J, Morris T, Stallard N, Tysall C, Willis A, Underwood M, the Repository G. Programme Grants for Applied Research. Identifying back pain subgroups: developing and applying approaches using individual patient data collected within clinical trials. Southampton (UK): NIHR Journals Library

Appendix 6: Subgroups identified by the recursive partitioning (RP) method for the sham vs non-active usual care comparison

| Subgroups | *n* | Treatment effect (95% confidence interval, CI) | Interaction effect | Unadjusted *p*-value |
| --- | --- | --- | --- | --- |
| ***Outcome: short-term MCS*** |  |  |  |  |
| ***Overall treatment effect (95% CI)***: 2.59 (1.13, 4.04) |  |  |  |  |
| *Candidate 1* |  |  |  |  |
| Age ≤ 65 | 705 | 3.4 (1.80, 5.04) | 4.32 | 0.019 |
| Age > 65 | 174 | -0.9 (-4.16, 2.35) |  |  |
|  |  |  |  |  |
| *Candidate 2* |  |  |  |  |
| PCS ≤ 42.0 | 791 | 3.1 (1.55, 4.65) | 4.99 | 0.043 |
| PCS > 42.0 | 88 | -1.9 (-6.07, 2.28) |  |  |
| *Note: The first row of each candidate subgroup is the selected subgroup with enhanced treatment effect.*  PCS, physical component scale of SF-12/36; MCS, mental component scale of SF-12/36. | | | | |

Reproduced with permission from: Patel S, Hee SW, Mistry D, Jordan J, Brown S, Dritsaki M, Ellard DR, Friede T, Lamb SE, Lord J, Madan J, Morris T, Stallard N, Tysall C, Willis A, Underwood M, the Repository G. Programme Grants for Applied Research. Identifying back pain subgroups: developing and applying approaches using individual patient data collected within clinical trials. Southampton (UK): NIHR Journals Library

| 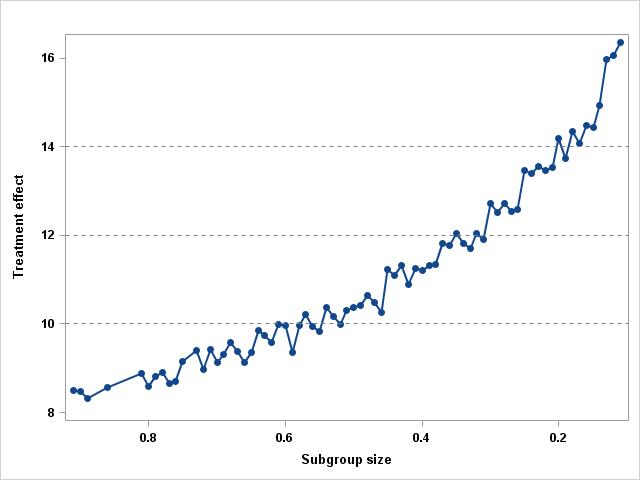  (a) *m* = 3; *n* = 3718 | 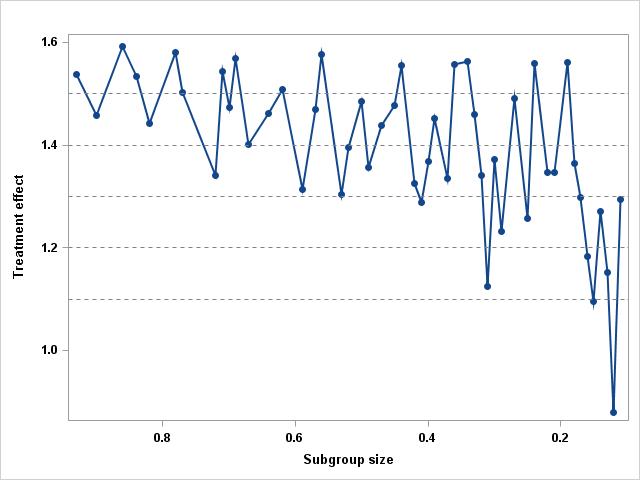  (b) *m* = 8; *n* = 2675 | 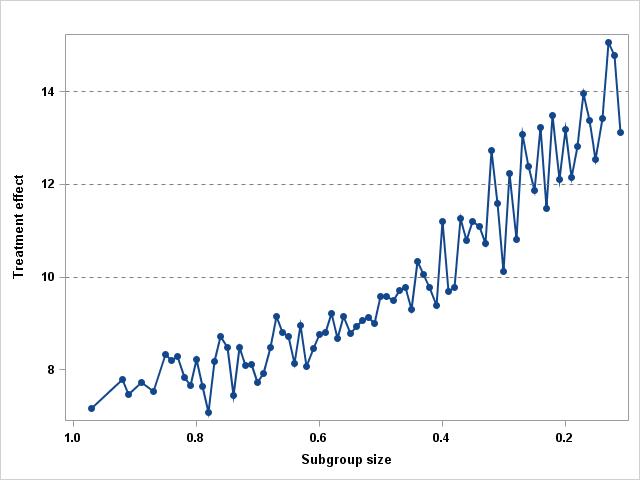  (c) *m* = 3; *n* = 2534 |
| --- | --- | --- |
| 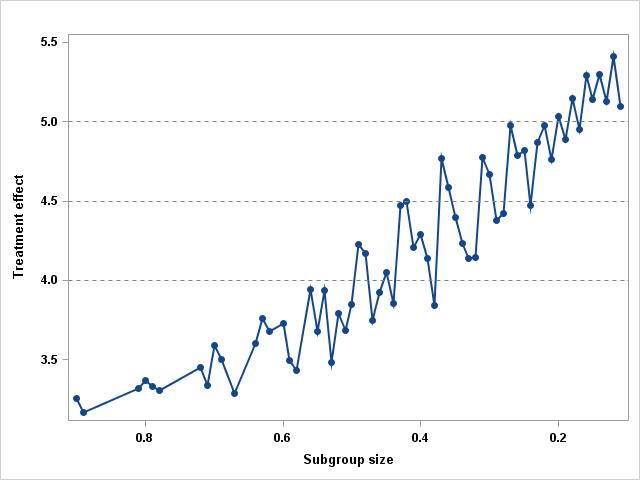  (d) *m* = 6; *n* = 5208 | 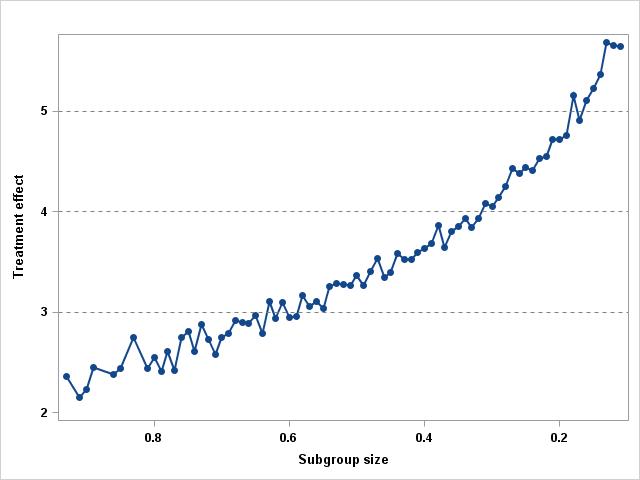  (e) *m* = 3; *n* = 3630 | 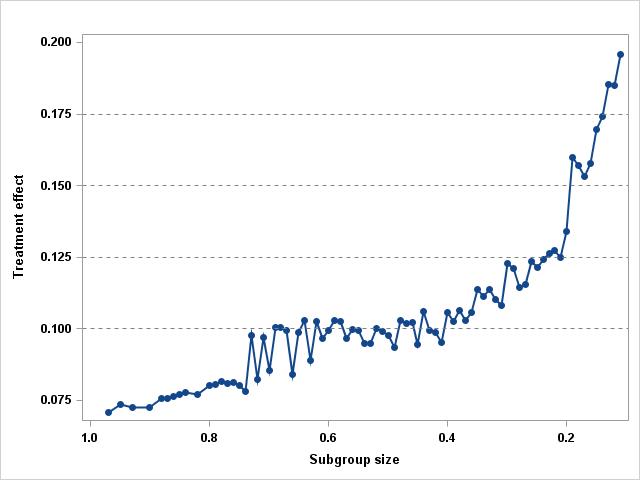  (f) *m* = 2; *n* = 1365 |
| **Appendix 7: Trajectory plot for the treatment effect against the size of the constructed region for short-term (a)**  **Hannover functional ability questionnaire for measuring back-pain related functional limitations, FFbHR, (b) Roland Morris disability questionnaire, RMDQ, (c) Pain, (d) physical component scale of SF-12/36, (e) mental component scale of SF-12/36, and (f) EQ-5D. The number of trials, *m*, and number of patients, *n*, in each of the subgroup identification analyses.** | | |

Adapted with permission from: Patel S, Hee SW, Mistry D, Jordan J, Brown S, Dritsaki M, Ellard DR, Friede T, Lamb SE, Lord J, Madan J, Morris T, Stallard N, Tysall C, Willis A, Underwood M, the Repository G. Programme Grants for Applied Research. Identifying back pain subgroups: developing and applying approaches using individual patient data collected within clinical trials. Southampton (UK): NIHR Journals Library

**Appendix 8: Thresholds for selected sizes of the subgroup for the short-term SF-12/36 MCS as seen in Appendix 7, figure (e).**

| **Subgroup size** | **Age (>)** | **PCS (<)** | **MCS (<)** | **FFbHR (<)** | **Treatment effect** |
| --- | --- | --- | --- | --- | --- |
| 0.108 | 16 | 28.84 | 50.61 | 100.00 | 5.98 |
| 0.207 | 31 | 43.62 | 50.61 | 45.83 | 4.72 |
| 0.301 | 54 | 43.62 | 56.82 | 75.00 | 4.05 |
| 0.401 | 45 | 67.75 | 60.35 | 62.50 | 3.64 |
| 0.505 | 31 | 38.01 | 60.35 | 75.00 | 3.37 |
| 0.604 | 45 | 67.75 | 60.35 | 100.00 | 2.94 |
| 0.701 | 16 | 47.59 | 60.35 | 75.00 | 2.75 |
| 0.807 | 16 | 47.59 | 60.35 | 100.00 | 2.55 |
| 0.907 | 16 | 47.59 | 72.11 | 100.00 | 2.23 |

FFbHR, Hannover functional ability questionnaire for measuring back-pain related functional limitations; PCS, physical component scale of SF-12/36; and MCS, mental component scale of SF-12/36.

Adapted with permission from: Patel S, Hee SW, Mistry D, Jordan J, Brown S, Dritsaki M, Ellard DR, Friede T, Lamb SE, Lord J, Madan J, Morris T, Stallard N, Tysall C, Willis A, Underwood M, the Repository G. Programme Grants for Applied Research. Identifying back pain subgroups: developing and applying approaches using individual patient data collected within clinical trials. Southampton (UK): NIHR Journals Library

**Appendix 9: Thresholds for selected sizes of the subgroup for the short-term EQ-5D as seen in Appendix 7, figure (f).**

| **Subgroup size** | **PCS (<)** | **MCS (<)** | **Pain (>)** | **RMDQ (>)** | **Treatment effect** |
| --- | --- | --- | --- | --- | --- |
| 0.101 | 35.66 | 60.35 | 0.00 | 14 | 0.21 |
| 0.190 | 31.34 | 72.11 | 0.00 | 6 | 0.16 |
| 0.210 | 33.62 | 56.82 | 0.00 | 6 | 0.13 |
| 0.303 | 40.45 | 47.17 | 0.00 | 6 | 0.12 |
| 0.407 | 67.75 | 72.11 | 57.00 | 0 | 0.11 |
| 0.505 | 40.45 | 72.11 | 30.00 | 6 | 0.10 |
| 0.593 | 47.59 | 56.82 | 0.00 | 6 | 0.10 |
| 0.610 | 67.75 | 56.82 | 20.00 | 6 | 0.10 |
| 0.704 | 47.59 | 60.35 | 20.00 | 5 | 0.09 |
| 0.803 | 47.59 | 60.35 | 0.00 | 0 | 0.08 |
| 0.909 | 67.75 | 60.35 | 0.00 | 0 | 0.07 |

RMDQ, Roland Morris disability questionnaire; PCS, physical component scale of SF-12/36; MCS, mental component scale of SF-12/36.

Adapted with permission from: Patel S, Hee SW, Mistry D, Jordan J, Brown S, Dritsaki M, Ellard DR, Friede T, Lamb SE, Lord J, Madan J, Morris T, Stallard N, Tysall C, Willis A, Underwood M, the Repository G. Programme Grants for Applied Research. Identifying back pain subgroups: developing and applying approaches using individual patient data collected within clinical trials. Southampton (UK): NIHR Journals Library
