# Supplementary material for: Identification of subgroup effect with an individual participant data meta-analysis of randomised controlled trials of three different types of therapist-delivered care in low back pain
Source: BMC Musculoskelet Disord. 2021 Feb 16;22:191. doi: 10.1186/s12891-021-04028-8 (PMC7885433; doi:10.1186/s12891-021-04028-8)
Supplement: Supplementary file 2 — Additional file 2: Table S1. One-step meta-analysis: Estimated difference between control (non-active usual care and sham) and all intervention treatments for each outcome adjusted by its baseline value for short-, mid-, and long-term follow-up. [file 12891_2021_4028_MOESM2_ESM.docx]

**Supplementary Table 1: One-step meta-analysis: Estimated difference between control (non-active usual care and sham) and all intervention treatments for each outcome adjusted by its baseline value for short-, mid-, and long-term follow-up.**

| **Outcome** | **Period** | ***m*** | ***n*_C_** | ***n*_I_** | **Estimated difference** | **95% confidence interval** | ***p*-value** |
| --- | --- | --- | --- | --- | --- | --- | --- |
| FFbHR (0 to 100; 100=best) | Short-term | 3 | 2118 | 1841 | 7.95 | (3.59 to 12.32) | 0.0156 |
|  | Mid-term | 3 | 2052 | 1807 | 3.88 | (-4.54 to 12.29) | 0.1860 |
| RMDQ (0 to 24; 24=worst) | Short-term | 8 | 897 | 1778 | 1.31 | (0.98 to 1.65) | <0.0001 |
|  | Mid-term | 5 | 474 | 798 | 1.51 | (1.04 to 1.99) | <0.0001 |
|  | Long-term | 7 | 741 | 1675 | 1.10 | (0.72 to 1.48) | <0.0001 |
| Pain* (0 to 100; 100=worst) | Short-term | 10 | 1546 | 2061 | 6.46 | (-1.60 to 14.53) | 0.0644 |
|  | Mid-term | 6 | 1144 | 1154 | 6.99 | (5.04 to 8.94) | <0.0001 |
|  | Long-term | 6 | 675 | 1623 | 4.76 | (2.51 to 7.00) | <0.0001 |
| PCS of SF-12/36 (0 to 100; 100=best) | Short-term | 6 | 2415 | 2793 | 3.16 | (1.95 to 4.38) | 0.0009 |
|  | Mid-term | 4 | 2057 | 1970 | 1.80 | (-0.74 to 4.33) | 0.1119 |
|  | Long-term | 4 | 582 | 1269 | 2.24 | (-0.26 to 4.74) | 0.0652 |
| MCS of SF-12/36 (0 to 100; 100=best) | Short-term | 6 | 2415 | 2793 | 1.66 | (0.56 to 2.77) | 0.0099 |
|  | Mid-term | 4 | 2057 | 1970 | 0.85 | (-0.89 to 2.58) | 0.1867 |
|  | Long-term | 4 | 582 | 1269 | 0.81 | (-0.63 to 2.26) | 0.2200 |
| EQ-5D (-0.11 to 1;1=best) | Short-term | 4 | 503 | 1271 | 0.039 | (0.015 to 0.064) | 0.0017 |
|  | Mid-term | 2 | 180 | 534 | 0.044 | (0.002 to 0.086) | 0.0415 |
|  | Long-term | 5 | 525 | 1363 | 0.039 | (0.015 to 0.064) | 0.0017 |
| Abbreviations: *m*, number of trials; *n*_C_, number of patients in the control arm; *n*_I_, number of patients in the intervention arm; short-, mid- and long-term follow-up, measurements taken 2 and 3 months, at 6 months and 12 months post randomization or entry to the trial, respectively; FFbHR, Hannover functional ability questionnaire for measuring back-pain related functional limitations; RMDQ, Roland Morris disability questionnaire; PCS, physical component scale of SF-12/36; MCS, mental component scale of SF-12/36.  * One of the following instruments from each trial, where available, was chosen (in descending order):  1. individual VAS on average pain today  2. average pain over the past 1 week  3. average pain over the past 2 weeks, average pain over the past 1 month  4. average pain over the past 3 months  5. the individual item of the CPG pain intensity score (CPG-PS) that is equivalent to the VAS if it is available  6. the summary score of the CPG-PS or  7. the bodily pain domain of SF-12/36. | | | | | | | |
