# Supplementary material for: Identification of subgroup effect with an individual participant data meta-analysis of randomised controlled trials of three different types of therapist-delivered care in low back pain
Source: BMC Musculoskelet Disord. 2021 Feb 16;22:191. doi: 10.1186/s12891-021-04028-8 (PMC7885433; doi:10.1186/s12891-021-04028-8)
Supplement: Supplementary file 3 — Additional file 3: Table S2. Moderator analysis for short-term outcomes (overall comparison). [file 12891_2021_4028_MOESM3_ESM.docx]

**Supplementary Table 2: Moderator analysis for short-term outcomes (overall comparison).**

| **Outcome** | **Covariates (baseline values)** | **Estimate (interaction term)** | **95% confidence interval** | ***p*-value** |
| --- | --- | --- | --- | --- |
| FFbHR | Age | -0.051 | (-0.131 to 0.028) | 0.2018 |
|  | FFbHR | -0.177 | (-0.229 to -0.125) | < 0.0001 |
|  | PCS | -0.318 | (-0.451 to -0.186) | < 0.0001 |
| RMDQ | Fear avoidance: Positive^a^ | 0.786 | (-0.125 to 1.697) | 0.0907 |
|  | Fear avoidance: Moderate^b^ | 0.714 | (-0.225 to 1.653) | 0.1361 |
|  | Catastrophising: Positive^c^ | 0.387 | (-2.271 to 3.046) | 0.7747 |
|  | Catastrophising: Moderate^d^ | 2.030 | (-0.461 to 4.521) | 0.1099 |
| Pain | Pain | 0.047 | (-0.017 to 0.111) | 0.1451 |
|  | PCS | -0.167 | (-0.400 to 0.066) | 0.1587 |
|  | MCS | 0.111 | (-0.047 to 0.268) | 0.1677 |
|  | Anxiety: low risk^e^ | -6.939 | (-15.111 to 1.233) | 0.0960 |
|  | Anxiety: moderate risk^f^ | -5.509 | (-14.423 to 3.405) | 0.2256 |
|  | Coping: Positive^g^ | -6.107 | (-14.999 to 2.786) | 0.1780 |
|  | Coping: Moderate^h^ | -2.864 | (-11.995 to 6.266) | 0.5382 |
|  | Fear avoidance: Positive | 1.396 | (-2.525 to 5.317) | 0.4851 |
|  | Fear avoidance: Moderate | 2.808 | (-1.031 to 6.646) | 0.1516 |
| PCS of SF-12/36 | Age | -0.034 | (-0.068 to 0.001) | 0.0538 |
|  | PCS | -0.057 | (-0.109 to -0.005) | 0.0313 |
|  | MCS (<50 vs. ≥50)^i^ | -0.913 | (-1.827 to 0.002) | 0.0504 |
| MCS of SF-12/36 | FFbHR | -0.046 | (-0.081 to -0.011) | 0.0093 |
|  | MCS (<50 vs. ≥50) | 1.490 | (0.442 to 2.539) | 0.0054 |
| EQ-5D | Sex (male vs. female)^j^ | -0.040 | (-0.094 to 0.015) | 0.1543 |
|  | RMDQ | 0.007 | (0.001 to 0.013) | 0.0219 |
|  | Pain | 0.002 | (0.000 to 0.003) | 0.0094 |
|  | PCS | -0.004 | (-0.008 to -0.001) | 0.0128 |
|  | MCS | -0.002 | (-0.004 to 0.001) | 0.1834 |
|  | Anxiety: low risk | -0.143 | (-0.232 to -0.055) | 0.0015 |
|  | Anxiety: moderate risk | -0.086 | (-0.180 to 0.009) | 0.0753 |
|  | Fear avoidance: Positive | -0.001 | (-0.072 to 0.071) | 0.9856 |
|  | Fear avoidance: Moderate | 0.073 | (-0.002 to 0.147) | 0.0565 |
| QALY | Age | 0.001 | (-0.0003 to 0.002) | 0.1850 |
|  | RMDQ | 0.003 | (-0.001 to 0.008) | 0.1270 |
|  | PCS | -0.001 | (-0.003 to 0.0004) | 0.1160 |

Abbreviations: FFbHR, Hannover functional ability questionnaire for measuring back-pain related functional limitations; RMDQ, Roland Morris disability questionnaire; PCS, physical component scale of SF-12/36; MCS, mental component scale of SF-12/36; QALY, quality-adjusted life-years.

a) estimate of the treatment effect for participants with positive belief (low fear avoidance) of fear avoidance belief was greater as opposed to those with the negative attitude; b) estimate of the treatment effect for participants with moderate belief of fear avoidance was greater as opposed to those with the negative attitude; c) estimate of the treatment effect for participants with positive attitude of catastrophising (low catastrophising score) was greater as opposed to those with the negative attitude (high catastrophising score); d) estimate of the treatment effect for participants with moderate attitude of catastrophising was greater as opposed to those with the negative attitude; e) estimate of the treatment effect for participants with low risk of anxiety was less as opposed to those with the high risk; f) estimate of the treatment effect for participants with moderate risk of anxiety was less as opposed to those with the high risk; g) estimate of the treatment effect for participants with positive attitude of coping strategy (high coping score) was less as opposed to those with the negative attitude (low coping score); h) estimate of the treatment effect for participants with moderate attitude of coping strategy was less as opposed to those with the negative attitude; i) estimate of the treatment effect for participants with SF-12/36 MCS score lower than general norm (<50) was less as opposed to those with score at or above the general norm (≥50); j) estimate of the treatment effect for male was less as opposed to female.
